# Implementation of the World Health Organization Global Antimicrobial Resistance Surveillance System in Uganda, 2015-2020: Mixed-Methods Study Using National Surveillance Data

**DOI:** 10.2196/29954

**Published:** 2021-10-21

**Authors:** Susan Nabadda, Francis Kakooza, Reuben Kiggundu, Richard Walwema, Joel Bazira, Jonathan Mayito, Ibrahimm Mugerwa, Musa Sekamatte, Andrew Kambugu, Mohammed Lamorde, Henry Kajumbula, Henry Mwebasa

**Affiliations:** 1 Laboratory and Diagnostics Services Department Ministry of Health Kampala Uganda; 2 Global Health Security Department Infectious Diseases Institute Kampala Uganda; 3 Department of Immunology and Molecular Biology Makerere University Kampala Uganda; 4 United States Agency for International Development Medicines, Technologies, and Pharmaceutical Services Program, Management Sciences for Health Kampala Uganda; 5 Department of Medical Microbiology Mbarara University of Science and Technology Mbarara Uganda; 6 Antimicrobial Resistance Sub-Committee National One Health Platform Kampala Uganda; 7 Department of Medical Microbiology Makerere University Kampala Uganda

**Keywords:** antimicrobial resistance, surveillance, microbiology, laboratory, Uganda, implementation, WHO, collection, analysis, data, antimicrobial, progress, bacteria, feasibility, resistance, antibiotic

## Abstract

**Background:**

Antimicrobial resistance (AMR) is an emerging public health crisis in Uganda. The World Health Organization (WHO) Global Action Plan recommends that countries should develop and implement National Action Plans for AMR. We describe the establishment of the national AMR program in Uganda and present the early microbial sensitivity results from the program.

**Objective:**

The aim of this study is to describe a national surveillance program that was developed to perform the systematic and continuous collection, analysis, and interpretation of AMR data.

**Methods:**

A systematic qualitative description of the process and progress made in the establishment of the national AMR program is provided, detailing the progress made from 2015 to 2020. This is followed by a report of the findings of the isolates that were collected from AMR surveillance sites. Identification and antimicrobial susceptibility testing (AST) of the bacterial isolates were performed using standard methods at both the surveillance sites and the reference laboratory.

**Results:**

Remarkable progress has been achieved in the establishment of the national AMR program, which is guided by the WHO Global Laboratory AMR Surveillance System (GLASS) in Uganda. A functional national coordinating center for AMR has been established with a supporting designated reference laboratory. WHONET software for AMR data management has been installed in the surveillance sites and laboratory staff trained on data quality assurance. Uganda has progressively submitted data to the WHO GLASS reporting system. Of the 19,216 isolates from WHO GLASS priority specimens collected from October 2015 to June 2020, 22.95% (n=4411) had community-acquired infections, 9.46% (n=1818) had hospital-acquired infections, and 68.57% (n=12,987) had infections of unknown origin. The highest proportion of the specimens was blood (12,398/19,216, 64.52%), followed by urine (5278/19,216, 27.47%) and stool (1266/19,216, 6.59%), whereas the lowest proportion was urogenital swabs (274/19,216, 1.4%). The mean age was 19.1 (SD 19.8 years), whereas the median age was 13 years (IQR 28). Approximately 49.13% (9440/19,216) of the participants were female and 50.51% (9706/19,216) were male. Participants with community-acquired infections were older (mean age 28, SD 18.6 years; median age 26, IQR 20.5 years) than those with hospital-acquired infections (mean age 17.3, SD 20.9 years; median age 8, IQR 26 years). All gram-negative (*Escherichia coli, Klebsiella pneumoniae*, and *Neisseria gonorrhoeae*) and gram-positive (*Staphylococcus aureus and Enterococcus* sp) bacteria with AST showed resistance to each of the tested antibiotics.

**Conclusions:**

Uganda is the first African country to implement a structured national AMR surveillance program in alignment with the WHO GLASS. The reported AST data indicate very high resistance to the recommended and prescribed antibiotics for treatment of infections. More effort is required regarding quality assurance of laboratory testing methodologies to ensure optimal adherence to WHO GLASS–recommended pathogen-antimicrobial combinations. The current AMR data will inform the development of treatment algorithms and clinical guidelines.

## Introduction

### Background

Antimicrobial resistance (AMR) is associated with increased morbidity and mortality and is recognized as an emerging global health threat. If left unchecked, by 2050, AMR may contribute up to 10 million deaths per year [1]. In low- and middle-income countries (LMICs), particularly in Africa, data on drug-resistant infections are extremely scarce [2]. A few available reports indicate that resistance to commonly prescribed antibiotics is prevalent, but the methodology for the generation and reporting of AMR data is suboptimal [3,4]. A systematic review targeting policy makers in East Africa found significant knowledge gaps in AMR and recommended strengthening antimicrobial stewardship and AMR surveillance in the region [5].

In Uganda, early efforts against AMR identified the critical gap as a lack of routine surveillance systems with limited data, making it difficult to track the AMR burden [6]. Moreover, a substantial proportion of methicillin-resistant *Staphylococcus aureus* and gram-negative organisms in different sample types has been reported [7]. Another study at a Ugandan regional referral hospital (RRH) on antimicrobial-resistant infections among postpartum mothers recommended increased microbiological testing [8]. This informs appropriate antibiotic use, development of antimicrobial stewardship programs, and strengthening of infection prevention and control practices as top priorities. Notably, through an ongoing sentinel surveillance program, microbiology capacity has been enhanced in selected RRHs that contribute significantly toward bacterial ID and antimicrobial susceptibility testing (AST) in Uganda [9]. In addition, Uganda is among the few African countries that have adopted and established a quality-assured World Health Organization (WHO) Enhanced Gonococcal Antimicrobial Surveillance Program and reported data locally and globally [10,11].

In 2015, the WHO launched the Global Laboratory AMR Surveillance System (GLASS) and initiated its implementation in the human health sector [12]. The GLASS program provides national guidance on AMR, focusing on different surveillance methods for adoption and priority specimens, pathogens, and pathogen-antibacterial combinations for use within national surveillance programs. GLASS enables monitoring of emerging AMR profiles at the country level and facilitates the development of hospital-based antibiograms to inform clinical treatment decisions.

In line with global calls to enhance support for AMR systems in LMICs, development partners are currently supporting Uganda’s national laboratory system and, more recently, the National Action Plan (NAP) for AMR [13] using a system-strengthening approach [14]. Since 2015, the United States with the help of the Centers for Disease Control and Prevention has been supporting the laboratory capacity at national and regional referral levels to enhance sample transportation systems for microbiology samples. In 2018, the Fleming Fund of the United Kingdom initiated support to the Government of Uganda to strengthen national coordination efforts for AMR using the One Health approach. The efforts are targeting to expand the microbiology testing capacity at the national level and selected RRHs and generate quality-assured AMR data. With the support of these and other partners, Uganda is implementing its NAP for AMR, which was formally launched in 2019.

### Objective

In this paper, we highlight the progress on the implementation of GLASS in Uganda from October 2015 to June 2020 and describe laboratory-based AMR surveillance data obtained from selected surveillance sites in the same period. The data include corresponding participant characteristics (sex and age), source of bacterial infection for surveillance hospital-acquired infections (HAIs), bacterial recovery rates, and resistance profiles.

## Methods

### Overview

A mixed methodology was used to obtain data presented in this study. Qualitative methods were used for the program setup, whereas quantitative methods were used to generate the AMR surveillance isolate data. A situational analysis report by the Uganda National Academy of Sciences was reviewed to understand the existing national AMR capacity and provide important information to guide program development [15]. To develop a sustainable national AMR surveillance program, the Ministry of Health (MoH) benchmarked on international guidance, using the approach first described in the WHO GLASS manual for the early implementation [12] and later interpreted according to the road map for participation in GLASS by Seale et al [16]. These recommendations were implemented under the cognizance of the local context of Uganda’s health systems. A systematic stepwise capacity-building approach [17] was used to set up and implement the program. The approach focused on setting up structures, systems, and roles at national and subnational levels; addressing staffing and infrastructure needs; and providing skills and tools to health workers. Stakeholders’ engagement was undertaken to ensure a supportive environment for the implementation of AMR surveillance. The partners supporting the national AMR surveillance program include the Centers for Disease Control and Prevention, the Fleming Fund, the World Bank, and academic institutions, including Makerere University and Mbarara University of Science and Technology. Using the outline by Seale et al [16], we describe the steps taken and the achievements of developing the national AMR surveillance program and later present the AMR data obtained from the program under the *Results* section.

### Key Achievements

#### Enrollment of Uganda in GLASS

In 2015, Uganda responded to the WHO call for countries to enroll in the GLASS program. By enrolling in the GLASS program, Uganda committed to collecting and sharing national AMR surveillance data. As part of this process, the country also acquired the WHONET software [18] used to report AMR surveillance data. A WHO GLASS focal person was designated by the MoH to support the coordination of AMR data validation, quality assurance, and the reporting process. Uganda now participates in the annual AMR data submissions to GLASS, and the country AMR data are part of the WHO global AMR surveillance reports [19].

#### Establishment of the AMR National Coordinating Center

The National Coordinating Center (NCC) at the MoH has been set up to oversee the national AMR surveillance program in human health, including the collection and aggregation of data from surveillance sites. The NCC works in collaboration with the Uganda National AMR Sub-Committee (UNAMRsC) of the One Health approach to provide strategic oversights of the national AMR program. The UNAMRsC has also been established as part of the governance structure for AMR in the country. The membership of the UNAMRsC also includes representation from other relevant line ministries, such as animal health, wildlife, and the environment. The mandate of the UNAMRsC includes defining the national AMR surveillance objectives; developing and disseminating protocols; coordinating data collection, analysis, and reporting; and reviewing data before reporting to GLASS. To date, the NCC has supported the development of key AMR surveillance documents, including national AMR surveillance plans, protocols, guidelines, curricula, and microbiology standard operating procedures.

#### Finalization of the NAP for AMR

The WHO requested all member countries to develop multisectoral-wide NAPs that are aligned with the Global Action Plan for AMR to support the implementation of the national AMR programs. Working with partners, the UNAMRsC completed the development of the Uganda AMR NAP [13], which was launched in November 2018 and now supports the implementation of priority activities in the NAP for AMR.

#### Designation of the National Microbiology Reference Laboratory

Initially, the Department of Medical Microbiology Laboratory at Makerere University was designated as the AMR Surveillance Laboratory. However, the capacity of the Central Public Health Laboratories has been built gradually, and it is now the designated national microbiology reference laboratory for AMR surveillance. The capacity built at Central Public Health Laboratories includes human resource development, quality management systems toward accreditation, isolate transportation, enhanced biorepository, and enrollment of laboratories in an External Quality Assurance scheme. In addition, the state-of-the-art Becton and Dickson–manufactured equipment, including matrix assisted laser desorption/ionization time of flight [20] and Phoenix M50 [21], has been installed at the reference laboratories and BACTEC blood culture systems, including FX 200 and FX40 [22], at selected RRH laboratories. These results support bacterial ID and AST.

#### Selection and Capacity Building for AMR Surveillance Sites

**Figure 1:**
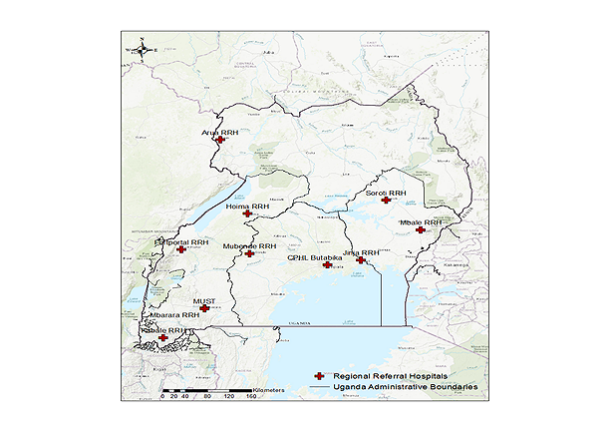
Geographic locations of the sentinel sites participating in the National Antimicrobial Resistance surveillance program.

In 2016, the AMR NCC designated different facilities as AMR surveillance sites. The sites were selected to ensure a balanced geographic, demographic, and socioeconomic distribution. They offer both outpatient and inpatient services, as per GLASS recommendations. However, the capacity of health facilities to conduct AMR surveillance varied between the different health facilities. As a result, selected sites have reported AMR surveillance data to the WHO and the capacity of the surveillance sites has been gradually developed. The Medicines and Therapeutics Committees oversee the implementation of the AMR surveillance program at the surveillance sites, which have been trained in collecting, analyzing, and reporting epidemiological, clinical, and laboratory data. The Medicines and Therapeutics Committee is usually headed by a senior consultant (Internal medicine, Gynecology, Surgery, and Pediatrics) who leads the stewardship of the AMR program at the site. On the basis of the clinician’s request for bacterial ID and AST as part of patient care, samples are collected according to the clinical protocols appropriate for the clinical presentation of patients and sent to the microbiology laboratory. The samples mainly include blood, urine, stool, and urogenital swabs and are accompanied by a microbiology laboratory request form that captures epidemiological information such as patient demographics and clinical presentation. The bacterial ID and AST data in this report were collected from 10 surveillance site microbiology laboratories between October 2015 and June 2020. The surveillance sites included Department of Medical Microbiology, Mbarara University of Science and Technology, Arua RRH, Kabale RRH, Mbarara RRH, Mubende RRH, Fort Portal RRH, Hoima RRH, Jinja RRH, Mbale RRH, and Soroti RRH (Figure 1). The surveillance sites have microbiology laboratories with the capacity to isolate, identify, and conduct microbial sensitivity testing for GLASS priority pathogens.

#### Bacterial ID and AST

In the laboratory, bacterial ID and AST for the different samples were collected, and subsequently isolates were performed in accordance with the standardized microbiology protocols and standard operating procedures. Blood culture vials were placed in a BACTEC 9050 or FX40 blood culture system (Becton-Dickinson) according to the manufacturer’s instructions. Samples from flagged positive vials were subjected to Gram staining and then cultured on blood agar, chocolate agar, and MacConkey agar culture plates. The culture plates were incubated for 18 to 24 hours at 35°C to 37°C, with recovered colonies undergoing conventional biochemical testing to confirm ID. For stool samples, a loop full of emulsified sample was inoculated on deoxycholate citrate agar, or Xylose Lysine Deoxycholate agar, and MacConkey agar and incubated at 35°C to 37°C in ambient air for 18 to 24 hours. Isolation and ID of growth was performed using conventional methods. Urine samples were gently mixed and inoculated on MacConkey agar and blood agar using an appropriate calibrated loop and incubated in ambient air overnight for 18 to 24 hours at 35°C to 37°C. Isolation, conventional ID, and colony counting were performed where applicable. Both urine and stool were examined macroscopically and microscopically (Gram staining). All urogenital swabs were inoculated on selective modified Thayer Martin and nonselective chocolate agar culture plates and then incubated at 35°C to 37°C in 5% CO_2_-enriched humid conditions. ID of gonococci colonies was based on the growth of the colonies with typical morphology in the modified Thayer Martin medium with a positive oxidase test [23]. AST was performed using the Kirby-Bauer disk diffusion method according to the Clinical and Laboratory Standards Institute [24]. We followed the WHO GLASS pathogen-antimicrobial combinations to set the antibiotics for susceptibility testing [12]. Preliminary culture results were immediately sent to the hospital wards to help clinicians optimize patient management, whereas the final results were shared later.

#### Submission of AMR Data for National and WHO GLASS Reporting

All laboratory results (bacterial ID and AST), patient demographics, and clinical data were entered into the microbiology register. These data were then entered into the WHONET software program [18] on a weekly basis for data analysis to generate facility-based AMR surveillance reports. The AMR surveillance subcommittee technical working committee representatives conducted data quality assessments on a quarterly basis to inform key performance indicators and reports submitted to NCCs. Uganda has been consistently submitting data to GLASS reporting since its enrollment in 2016 [19].

#### Statistical Analysis

Summary statistics were calculated for key demographic characteristics of participants from whom samples were collected and stratified by the origin of the samples. Age was summarized as a continuous variable and categorized with age groups defined. *P* values based on chi-square tests were calculated for each variable to provide a sense of differences in demographic characteristics among different origins. For each specimen type, the percentage recovery for all bacterial pathogens and GLASS priority pathogens was expressed as the proportion of samples with a positive culture result out of the total samples cultured. Similarly, for each pathogen, resistance was expressed as the proportion of isolates with resistant or intermediate results out of the total number of isolates tested for susceptibility to a specific antibiotic. The binomial 95% CIs for the proportions of recovery and resistance were calculated using the Wilson method. The frequency of infection with resistant pathogens could not be calculated because data on the population at risk were unavailable. In addition, because of potential sampling bias, no statistical analysis was performed to identify any associations or risk factors for the occurrence of resistant pathogens. The analysis was performed using Excel (Microsoft), R version 3.6 (R Foundation for Statistical Computing), and WHONET.

## Results

### Demographics of Participants

Of the 19,216 participants involved in the surveillance program, 22.95% (4411/19,216) had community-acquired infections, 9.46% (1818/19,216) had HAIs, and 68.57% (12,987/19,216) had infections of unknown origin (Table 1). The mean age was 19.1 years (SD 19.8 years), whereas the median was 13 years (IQR 28 years). Approximately 49.13% (9440/19,216) of the participants were female, and 50.51% (9706/19,216) were male. Participants with community-acquired infection were older (mean age 28 years, SD 18.6 years; median age 26 years, IQR 20.5 years) than those with HAIs (mean age 17.3 years, SD 20.9 years; median age 8 years, IQR 26 years).

**Table 1:** Characteristics of participants in the antimicrobial resistance surveillance program from 10 surveillance sites from October 2015 to June 2020.

Characteristics			Origin							Total (N=19,216)			*P* value	
			Community acquired (n=4411)		Hospital acquired (n=1818)		Unknown (n=12,987)							
Value, mean (SD)			28.0 (18.6)		17.3 (20.9)		15.9 (19.0)		19.1 (19.8)			N/A^a^		
Value, median (IQR)			26 (20.5)		8 (26)		6 (27)		13 (28)			N/A		
**Age (years), n (%)**														<.001
	<1	230 (5.21)		192 (10.56)		1753 (13.49)		2175 (11.32)						
	1-4	365 (8.27)		485 (26.68)		3003 (23.12)		3853 (20.05)						
	05-14	372 (8.43)		459 (25.25)		1600 (12.32)		2431 (12.65)						
	15-24	909 (20.61)		149 (8.19)		1042 (8.02)		2100 (10.92)						
	25-34	1083 (24.55)		168 (9.24)		1267 (9.76)		2518 (13.1)						
	35-44	572 (12.97)		120 (6.6)		748 (5.76)		1440 (7.49)						
	45-54	350 (7.93)		88 (4.84)		463 (3.56)		901 (4.69)						
	55-64	170 (3.85)		37 (2.04)		268 (2.06)		475 (2.47)						
	65-80	149 (3.38)		60 (3.3)		229 (1.76)		438 (2.28)						
	>81	52 (1.18)		30 (1.65)		51 (0.39)		133 (0.69)						
	Unknown	159 (3.6)		30 (1.65)		2563 (19.74)		2752 (14.32)						
**Sex, n (%)**														<.001
	Female	2413 (54.72)		836 (45.98)		6191 (47.67)		9440 (49.13)						
	Male	1974 (44.75)		980 (53.91)		6752 (51.99)		9706 (50.51)						
	Unknown	24 (0.54)		2 (0.11)		44 (0.34)		70 (0.36)						
**Facility, n (%)**														<.001
	Arua RRH^b^	1026 (23.26)		384 (21.12)		1049 (8.08)		2459 (12.79)						
	DMM MUST^c^	480 (10.88)		35 (1.92)		2351 (18.13)		2866 (14.91)						
	Fort Portal RRH	390 (8.84)		270 (14.85)		99 (0.76)		759 (3.95)						
	Hoima RRH	7 (0.16)		2 (0.11)		164 (1.26)		173 (0.9)						
	Jinja RRH	88 (1.99)		336 (18.48)		4398 (33.86)		4822 (25.09)						
	Kabale RRH	699 (15.84)		106 (5.83)		2191 (16.87)		2996 (15.59)						
	Mbale RRH	653 (14.8)		163 (8.97)		692 (5.33)		1508 (7.85)						
	Mbarara RRH	392 (8.89)		277 (15.24)		132 (1.02)		801 (4.17)						
	Mubende RRH	312 (7.07)		153 (8.42)		1464 (11.27)		1929 (10.04)						
	Soroti RRH	364 (8.25)		92 (5.06)		447 (3.44)		903 (4.69)						
**Department, n (%)**														<.001
	Inpatient	1186 (26.89)		1509 (83)		4527 (34.86)		7222 (37.58)						
	Outpatient	2850 (64.61)		181 (9.96)		3289 (25.32)		6320 (32.89)						
	Unknown	375 (8.5)		128 (7.04)		5171 (39.82)		5674 (29.53)						

^a^N/A: not applicable.

^b^RRH: regional referral hospital.

^c^DMM MUST: Department of Medical Microbiology, Mbarara University of Science and Technology.

A total of 19,216 WHO GLASS priority specimens were collected for microbiological testing from 10 surveillance sites over a period of 4 years and 6 months, from October 2015 to June 2020. The highest proportion of the specimens was blood (12,398/19,216, 64.52%), followed by urine (5278/19,216, 27.47%) and stool (1266/19,216, 6.59%), whereas the lowest proportion was that of urogenital swabs (274/19,216, 1.43%).

### Recovery Rates and Distribution of Pathogens

The overall recovery rate of the GLASS priority pathogens from the GLASS priority specimens was 7.4% (1429/19,216; Table 2). The recovery rates from the different samples were as follows: urogenital swabs 17.9% (49/274), urine 12.1% (637/5278), stool 7.74% (98/1266), and blood 5.2% (645/12,398). The highest percentage of GLASS priority pathogens identified were *Escherichia coli* (652/1429, 45.62%), followed by *S aureus* (337/1429, 23.58%), with the lowest being *Acinetobacter baumannii* (6/1429, 0.42%).

**Table 2:** Bacterial recovery rates from priority specimens collected from 10 surveillance sites, October 2015 to June 2020.

Variable			Value, n (%)		Odds ratio (95% CI)
**Samples cultured (n=19,216)**					N/A^a^
	Blood	12,398 (64.52)			
	Urogenital swabs	274 (1.43)			
	Stool	1266 (6.59)			
	Urine	5278 (27.47)			
**Samples with bacterial growth (n=4471)**					23.3 (22.7-23.9)
	Blood	1520 (33.99)		12.3 (11.7-12.9)	
	Urogenital swabs	174 (3.89)		63.5 (57.7-69.0)	
	Stool	491 (10.98)		38.8 (36.1-41.5)	
	Urine	2286 (51.13)		43.3 (42.0-44.6)	
**Samples yielding the GLASS^b^ priority pathogens (n=1429)**					7.4 (7-7.8)
	Blood	645 (45.14)		5.2 (4.8-5.6)	
	Urogenital swabs	49 (3.43)		17.9 (13.8-22.9)	
	Stool	98 (6.86)		7.7 (6.3-9.3)	
	Urine	637 (44.58)		12.1 (11.3-13.0)	
**GLASS priority pathogens recovered (n=1429)**					N/A
	*Escherichia coli*	652 (45.62)			
	*Staphylococcus* *aureus*	337 (23.58)			
	*Salmonella spp*	237 (16.58)			
	*Klebsiella* *pneumoniae*	109 (7.63)			
	*Neisseria gonorrhoeae*	49 (3.43)			
	*Shigella spp*	21 (1.47)			
	*S pneumoniae*	18 (1.26)			
	*Acinetobacter baumannii*	6 (0.42)			

^a^N/A: not applicable.

^b^GLASS: Global Laboratory Antimicrobial Resistance Surveillance System.

### Pathogen Resistance

Resistance patterns for the most commonly isolated gram-negative bacteria, that is, *E coli, Neisseria gonorrhoeae, Shigella* sp*,* and *Salmonella* sp are shown in Table 3, with all gram-negative bacteria showing resistance to each of the tested antibiotics. High resistance of *E coli* was noted among commonly used antibiotics, with 52.7% (95% CI 46.8%-58.5%) resistance to ceftriaxone, 18.8% (95% CI 14.9%-23.4%) resistance to imipenem, and 52% (95% CI 47.5%-56.6%) resistance to ciprofloxacin. High resistance of *Klebsiella pneumoniae* was noted among the commonly used antibiotics. High resistance of *N gonorrhoeae* was noted among the commonly used antibiotics, with 10% (95% CI 1.8%-40.4%) resistance to ceftriaxone and 71.4% (95% CI 45.4%-88.3%) resistance to ciprofloxacin. High resistance of *Salmonella* and *Shigella* was noted among the commonly used antibiotics, including resistance to meropenem, ceftriaxone, and ciprofloxacin.

**Table 3:** Antimicrobial resistance profiles of selected gram-negative bacteria from 10 surveillance sites from October 2015 to June 2020.

Bacteria name	Antibiotic name	Number	R+I^a^ (95% CI; %)
*Escherichia coli*			
	Amikacin	52	7.7 (3-18.2)
	Amoxicillin	25	88 (70-95.8)
	Amoxicillin and clavulanic acid	232	75 (69.1-80.1)
	Ampicillin	359	91.6 (88.3-94.1)
	Cefoxitin	27	25.9 (13.2-44.7)
	Ceftazidime	77	45.5 (34.8-56.5)
	Ceftriaxone	277	52.7 (46.8-58.5)
	Cefuroxime	334	63.8 (58.5-68.7)
	Chloramphenicol	366	42.1 (37.1-47.2)
	Ciprofloxacin	455	52.1 (47.5-56.6)
	Clindamycin	54	88.9 (77.8-94.8)
	Erythromycin	100	92 (85-95.9)
	Gentamicin	403	38.2 (33.4-42.8)
	Imipenem	324	18.8 (14.9-23.4)
	Levofloxacin	35	5.7 (1.6-18.6)
	Meropenem	42	19 (10-33.3)
	Nalidixic acid	181	76.8 (70.1-82.3)
	Nitrofurantoin	266	30.8 (25.6-36.6)
	Penicillin G	71	97.2 (90.3-99.2)
	Piperacillin or tazobactam	33	36.4 (22.2-53.4)
	Tetracycline	226	78.8 (73-83.6)
	Trimethoprim-sulfamethoxazole	327	83.8 (79.4-87.4)
	Vancomycin	94	76.6 (67.1-84)
*Klebsiella* *pneumoniae*			
	Amoxicillin	50	86 (73.8-93)
	Ampicillin	79	97.5 (91.2-99.3)
	Ceftazidime	20	65 (43.3-81.9)
	Ceftriaxone	79	79.7 (69.6-87.1)
	Cefuroxime	63	77.8 (66.1-86.3)
	Chloramphenicol	78	53.8 (42.9-64.5)
	Ciprofloxacin	86	53.5 (43-63.7)
	Gentamicin	78	71.8 (61-80.6)
	Imipenem	63	1.6 (0.3-8.5)
	Meropenem	21	23.8 (10.6-45.1)
	Tetracycline	42	54.8 (39.9-68.8)
	Trimethoprim-sulfamethoxazole	67	82.1 (71.3-89.4)
*Neisseria gonorrhoeae*			
	Ceftriaxone	10	10 (1.8-40.4)
	Cefuroxime	13	46.2 (23.2-70.9)
	Ciprofloxacin	14	71.4 (45.4-88.3)
	Tetracycline	16	100 (80.6-100)
*Salmonella sp*			
	Amikacin	37	5.4 (1.5-17.7)
	Amoxicillin	33	100 (89.6-100)
	Ampicillin	131	81.7 (74.2-87.4)
	Ceftazidime	66	13.6 (7.3-23.9)
	Ceftriaxone	98	17.3 (11.1-26)
	Cefuroxime	108	20.4 (13.9-28.9)
	Chloramphenicol	138	66.7 (58.4-74)
	Ciprofloxacin	116	24.1 (17.3-32.7)
	Gentamicin	52	17.3 (9.4-29.7)
	Imipenem	83	3.6 (1.2-10.1)
	Levofloxacin	60	1.7 (0.3-8.9)
	Nalidixic acid	127	15.7 (10.4-23.1)
	Tetracycline	96	87.5 (79.4-92.7)
	Trimethoprim-sulfamethoxazole	114	69.3 (60.3-77)
*Shigella sp*			
	Amikacin	15	93.3 (70.2-98.8)
	Ceftriaxone	19	15.8 (5.5-37.6)
	Cefuroxime	10	50 (23.7-76.3)
	Chloramphenicol	11	54.5 (28-78.7)
	Ciprofloxacin	20	30 (14.5-51.9)
	Gentamicin	13	23.1 (8.2-50.3)
	Nalidixic acid	12	33.3 (13.8-60.9)
	Tetracycline	12	50 (25.4-74.6)
	Trimethoprim-sulfamethoxazole	13	38.5 (17.7-64.5)

^a^R+I: Resistance + Intermediate.

Among gram-positive bacteria, high resistance of *S aureus* was noted among the commonly used antibiotics, with 42.9% (95% CI 28%-59.1%) resistance to cefoxitin, 30.9% (95% CI 21.2%-42.6%) resistance to oxacillin, 76.9% (95% CI 69%-83.2%) resistance to TMP-SMX, and 15.5% (95% CI 9.6%-24%) resistance to vancomycin (Table 4). High resistance of *Enterococcus* sp was noted among the commonly used antibiotics, with 81.8% (95% CI 61.5%-92.7%) resistance to ciprofloxacin and 50% (95% CI 33.6%-66.4%) resistance to vancomycin. A high resistance of *Streptococcus* sp was noted between vancomycin and ceftriaxone.

**Table 4:** Antimicrobial resistance profiles of selected gram-positive bacteria from 10 surveillance sites, from October 2015 to June 2020.

Bacteria name	Antibiotic name	Number	R+I^a^ (95% CI; %)
*Staphylococcus* *aureus*			
	Amoxicillin and clavulanic acid	47	51.1 (37.2-64.7)
	Ampicillin	54	81.5 (69.2-89.6)
	Cefoxitin	35	42.9 (28-59.1)
	Ceftazidime	57	12.3 (6.1-23.2)
	Ceftriaxone	74	41.9 (31.3-53.3)
	Cefuroxime	93	20.4 (13.5-29.7)
	Chloramphenicol	176	56.2 (48.9-63.4)
	Ciprofloxacin	161	41 (33.7-48.7)
	Clindamycin	120	16.7 (11.1-24.3)
	Erythromycin	181	68 (60.8-74.3)
	Gentamicin	158	31 (24.3-38.6)
	Imipenem	101	13.9 (8.4-21.9)
	Levofloxacin	61	0 (0-5.9)
	Moxifloxacin	43	0 (0-8.2)
	Ofloxacin	61	0 (0-5.9)
	Oxacillin	68	30.9 (21.2-42.6)
	Penicillin G	106	86.8 (79-92)
	Tetracycline	162	72.2 (64.9-78.5)
	Trimethoprim-sulfamethoxazole	134	76.9 (69-83.2)
	Vancomycin	97	15.5 (9.6-24)
*Enterococcus sp*			
	Ampicillin	32	87.5 (71.9-95)
	Chloramphenicol	21	61.9 (40.9-79.2)
	Ciprofloxacin	22	81.8 (61.5-92.7)
	Erythromycin	35	91.4 (77.6-97)
	Gentamicin	14	57.1 (32.6-78.6)
	Tetracycline	15	73.3 (48-89.1)
	Vancomycin	32	50 (33.6-66.4)
	Gentamicin-high	14	64.3 (38.8-83.7)
*Streptococcus sp*			
	Ceftriaxone	14	64.3 (38.8-83.7)
	Chloramphenicol	45	35.6 (23.2-50.2)
	Clindamycin	49	32.7 (21.2-46.6)
	Erythromycin	52	59.6 (46.1-71.8)
	Penicillin G	15	66.6 (41.7-84.8)
	Tetracycline	29	51.7 (34.4-68.6)
	Trimethoprim-sulfamethoxazole	20	75 (53.1-88.8)
	Vancomycin	24	25 (12-44.9)

^a^R+I: Resistance + Intermediate.

## Discussion

### Principal Findings

The findings of our surveillance program show the feasibility of setting up a national AMR surveillance program based on the WHO GLASS manual recommendation while building systems for quality assurance, data sharing, linking results to patient care, and building partnerships. AMR is a global health threat, and the establishment of national surveillance systems is necessary to identify the emerging drug-resistant infections [2]. The African region still has suboptimal microbiology laboratory capacity and surveillance systems for AMR [3]. However, in Africa and Uganda in particular, resistance to recommended antibiotics has been reported for the WHO GLASS priority pathogens [12]. This paper presents the processes undertaken to set up a national AMR surveillance system according to WHO GLASS standards in Uganda, which could be benchmarked for other LMICs. The surveillance sites were RRHs and an academic institution. The RRHs represented the majority of the geographical distribution as they received referrals from district hospitals and health centers IVs and IIIs.

The success of establishing the national AMR surveillance program in Uganda highlights the feasibility of implementing the WHO GLASS program in LMICs. AMR surveillance programs are fundamental in Sub-Saharan African countries such as Uganda for generating antibiograms that can inform the development of treatment guidelines and antibiotic procurement plans and contribute toward standardized reporting [25,26]. Clinicians at surveillance sites can also access bacterial ID and AST results to inform patient care because of the availability of strengthened quality microbiology services.

In Uganda, the AMR surveillance system has been established using a systematic capacity-building pyramid model [17] and in alignment with the London School of Hygiene and Tropical Medicine stepwise road map for participating in GLASS [16]. The rolling out of the NAP for AMR [13], AMR national and subnational structures with Terms of Reference, and supporting surveillance plans and protocols has strengthened antimicrobial stewardship. In addition, the establishment of data-sharing platforms, including software programs such as WHONET [18], has supported data collation, analysis, reporting, and electronic archival, supplementing the existing paper-based methods.

The Uganda surveillance program identified selected resistant priority pathogens, including *E coli*, *S aureus*, *K pneumoniae*, and *N gonorrhoeae*, which present a high diversity of pathogens seen in Central Africa, Gabon [27]. *E coli* and *S aureus* isolates were the most prevalent WHO priority pathogens isolated in Uganda. This is fundamental baseline information that could be used for pretesting the novel WHO protocol [28] for estimating mortality attributable to AMR bloodstream infections. Monitoring priority pathogens and analyzing their antimicrobial susceptibilities together with epidemiological information on sex, age, and surveillance site can inform early hospital-level interventions [29].

There was a high rate of resistance of *E coli* to ampicillin and cotrimoxazole, as recently reported [3]. However, proportion of ceftriaxone- and ciprofloxacin-resistant *E coli* was slightly lower than that observed in Equatorial Guinea [30]. More worryingly, there was a significant proportion 18.8% (61/324) of *E coli* resistance to imipenem, which is considerably higher than 3%, recently reported in other parts of the African continent [3]. For *K pneumoniae,* there was notable resistance to ceftriaxone at 79% (63/79) and cotrimoxazole at 82% (55/67) in the surveillance program, similar to findings in Uganda’s neighboring country Kenya [31].

To our knowledge, this is the first documentation of the implementation of a national AMR surveillance program on the African continent using the WHO-recommended methodology. Although all components of the WHO GLASS manual are implemented, the approaches were not sequential and were contextualized to Uganda cognizance of existing national policies and programs.

The main limitations included suboptimal recovery of the AMR GLASS priority pathogens and inconsistent setting of the recommended antibiotics against the pathogen in the laboratory. This was attributed to the fact that staff members were still undergoing comprehensive training on microbiology skills, stock-outs, and acquiring extensive knowledge on AMR surveillance [32]. The number of samples sent to the microbiology laboratory was relatively low, coupled with low rates of completion of the microbiology laboratory request forms. This was partially attributed to the lack of a laboratory-clinician interface to bridge these anomalies.

### Conclusions

Using the WHO guidance, Uganda has successfully completed key foundational building activities for the successful implementation of a national AMR surveillance program. The emerging antibiotic resistance data can be refined, appraised, and used for further improvement of the current methodological approaches being used to implement AMR programs in Uganda and other LMICs. Uganda successfully enrolled in the WHO GLASS system and has consistently reported annual program progress to the WHO since 2016. There is an extremely high prevalence of AMR to the most commonly used antibiotics, similar to what has been found in other studies conducted in the research context.

### Recommendations

The current Uganda Clinical Guidelines need to be reviewed in response to the AMR burden in Uganda. In addition, strengthening the capacity of the microbiology laboratory is fundamental for the successful implementation of surveillance protocols in hospital wards to further profile the emerging global health threat of AMR.

## Abbreviations

AMR: antimicrobial resistance
AST: antimicrobial susceptibility testing
GLASS: Global Laboratory Antimicrobial Resistance Surveillance System
HAI: hospital-acquired infection
LMIC: low- and middle-income country
MoH: Ministry of Health
NAP: National Action Plan
NCC: National Coordinating Center
RRH: regional referral hospital
UNAMRsC: Uganda National Antimicrobial Resistance Sub-Committee
WHO: World Health Organization
